# Lung ultrasound can differentiate *Pneumocystis jiroveci *versus other etiologies among critically ill AIDS patients with pneumonia

**DOI:** 10.1186/cc10693

**Published:** 2012-03-20

**Authors:** A Japiassu, F Bozza

**Affiliations:** 1 IPEC-FIOCRUZ, Rio de Janeiro, Brazil

## Introduction

Lung ultrasound (US) can be applied as a point-of-care approach for diagnosis of pneumonia in AIDS patients. We compare US examinations of *Pneumocystis jiroveci *versus other etiologies of pneumonia in critically ill patients.

## Methods

Every HIV/AIDS patients admitted to the ICU with pneumonia was included. The first US examination was performed until 72 hours after admission. Pneumonia was defined by clinical examination, laboratorial parameters and chest X-rays. Etiologic agents were defined according to appropriate cultures and serology. US was applied to four fields (apex, lateral middle third, anterior basal and posterior basal regions) for each hemithorax, with 2.5 MHz curved transducer. Three pneumonia patterns were defined: interstitial pneumonia, bronchopneumonia and pneumonia with consolidation. The presence of B lines, peripheral microabscesses (bronchopneumonia), consolidations and pleural effusions were compared between the Pneumocystis pneumonia group (PCP) versus other etiologies.

## Results

We included 21 patients (age (median) 38 years; male 71%). Most (80%) patients were admitted because of acute respiratory insufficiency by pneumonia. Seventeen (81%) had CD4 cell counts lower than 200/mm^3^. The SAPS 2 score was 47 points and the SOFA score on day 1 of admission was 6 points. Hospital mortality was 43%. All radiographic pneumonia images were viewed on lung US examinations. Possible and probable pneumonia by *P. jiroveci *was diagnosed in six patients; all of these patients presented diffuse thin and/or gross B lines on both lungs. Bacterial (*n *= 7), mycobacterial (tuberculosis (*n *= 6) and *Mycobacterium kansasii *(*n *= 1)), and fungal (Aspergillus sp. (*n *= 1)) were diagnosed in other patients. Peripheral microabscesses were viewed on one patient with PCP and four patients with other etiologies (*P *= NS); pleural effusions were present on US of seven patients with diverse etiologies (no PCP patient had pleural effusions; *P *= 0.06); no pneumothorax was diagnosed in the study. Consolidation was present in one patient with PCP and 11 patients with bacterial, mycobacterial and fungal pneumonia (*P *= 0.05). There was a high degree of symmetry on lung US examinations of PCP patients, while there was always differences between the right and left hemithorax among other etiologic pneumonia (*P *< 0.001).

## Conclusion

We suggest that high-degree symmetric and diffuse B lines, without pleural effusions, are compatible with *P. jiroveci *as the etiology of recent diagnosed pneumonia in critically ill AIDS patients.

